# Aneurysm

**Published:** 1908-04-04

**Authors:** 


### Points in Surgery.

### ANEURYSM.

### SOME FACTORS IN ITS CAUSATION.

One frequently hears it said by surgeons that aneurysms are of far less common occurrence than they used to be. As far as aneurysms of the peripheral vessels are concerned, we must allow this to be the case, when we reflect that this used to be one of the commonest surgical ailments, and that not so very long ago, whereas nowadays an aneurysm of a large peripheral artery is regarded in the light of a surgical curiosity.

The word aneurysm is derived from the Greek εὔρυσμα a widening and ἀνα in the course of [an artery]. It may be defined as a blood tumour communicating with an artery. It is the outcome of a combination of causes. Of these the most important is a degeneration of the arterial wall. Syphilis is a prominent factor in producing these degenerative changes. It causes typically an endarteritis, particularly in the cerebral arteries, and so leads to aneurysm. This, of course, may be the result of increased blood pressure, or the direct outcome of weakening of the vessel wall. There is a tendency among pathologists to-day to regard the results of syphilitic infection as more far reaching than this. and it has been suggested that it also attacks and weakens the middle coat. If this is the case, it is easy to explain the disappearance of aneurysms on the ground that syphilis is better and more scientifically treated. Whether this is the cause or whether it is due to the fact that the population is becoming immunised against the disease by inherited taint from previous generations, there can be little doubt that syphilis is gradually dying out, or at any rate that it is far less virulent in its manifestations than was formerly the case.

Atheroma, too, is a condition which is said to dispose to aneurysm. Here again the exact method of causation is not clear. Some authorities are inclined to believe that atheroma is a process identical with arterio-capillary fibrosis; the only difference being that the former attacks large vessels and the latter smaller ones. German writers are so convinced of the connection between the two that they go so far as to use the term athero-sclerosis. This hardly simplifies the question, and does not explain the fact that arterio-capillary fibrosis affects the middle coat, whereas atheroma begins essentially in the sub-endothelial tissue of a large artery in the neighbourhood of one of its vasa vasorum, with a proliferation of connective tissue cells which subsequently degenerate and form a pulpy mass. Hence the term atheroma, a Greek word, whose literal meaning is "porridge." Sooner or later the endothelium over these collections gives way, and the result is an atheromatous ulcer; and this may or may not become calcified subsequently, so that the site of the ulcer is occupied by a calcareous plate. To discuss such a question may appear to be an intrusion into the parish of the physician, but it seems more reasonable to the writer to suppose that atheroma may be a result of acute aortitis. In this condition we see localised patches of degeneration of the endothelial coat and sub-endothelial tissue; and the distribution of these is much the same as that of atheromatous patches. The stumbling-block in the way of this argument is the comparative rarity of acute aortitis as opposed to the relative frequency of atheroma. Where one has so little definite knowledge and so little chance of investigating cases to one's satisfaction, one is bound to fall back on conjecture, and in some quarters an entirely different hypothesis has been put forward to explain the connection between the two—namely, that acute aortitis, when it occurs, does so in a vessel which is already atheromatous. This idea is based on the observed fact that the vascular endothelium is not easily vulnerable, and that acute infective endocarditis generally occurs in a heart already damaged by chronic disease.

Whatever the causation of atheroma, it certainly predisposes to aneurysm, either by causing a weak spot in the arterial wall which allows it to yield to the blood pressure, or by admitting the blood into the sub-endothelial tissues, and so causing a dissecting aneurysm, though this is a rare condition. Again, anything which deprives the vessel of its normal support from surrounding tissues disposes to aneurysmal dilatation. A good example of this is seen in pulmonary phthisis. When a cavity forms in the lung, it is traversed by the arteries of the part which are resistant structures as far as the tubercle bacillus is concerned. Small aneurysms make their appearance, and it is the rupture of these which causes the hæmoptysis of the later stages of pulmonary tuberculosis. In the domain of surgery a similar example is afforded by the popliteal artery. Aneurysm of this vessel is not at all uncommon; a fact which may be explained on anatomical grounds, for the popliteal artery lies practically unsupported in a wide space, where it is surrounded only by areolar connective tissue.

In addition to degenerative changes in, and abnormal conditions of, the vessel wall, any condition which increases the blood pressure must be regarded as an important ætiological factor; and here again one encroaches upon the territory of the physician, for most of these states are met with in the course of medical diseases. Thus chronic nephritis increases the blood pressure in two ways by causing (*a*) peripheral arterio-sclerosis and (*b*) cardiac hypertrophy. So also does sudden or continued exertion. A sudden exertion may cause an overaction of the heart and local damage to the arterial wall. In a case seen recently a man had an axillary aneurysm. No cause could be found for it, unless it was due to his having run after a motor omnibus some time previously. He caught hold of the rail of the omnibus, but it went on again before he had mounted it, and caused a severe wrench of the left arm, which had been painful ever since. Continued exertion may produce the same result, and more than one case of popliteal aneurysm has been recorded which has been attributed to a long bicycle tour undertaken by a patient who was not fitted to stand the strain.

